# Anesthetic Management in Familial Hyperkalemic Periodic Paralysis: A Case Report

**DOI:** 10.7759/cureus.19889

**Published:** 2021-11-25

**Authors:** Ali Atoot, Monica Paganessi, Michael Block, Mark D Schlesinger

**Affiliations:** 1 Anesthesiology, Hackensack University Medical Center, Hackensack, USA

**Keywords:** potassium-associated paralysis, anesthetic management, paralysis, general anesthesia successfully, flaccid weakness, hyperkalemic periodic paralysis

## Abstract

Hyperkalemic periodic paralysis (HyperPP) is the rarer of two forms of potassium-associated familial paralysis characterized by episodic flaccid weakness secondary to an increase in serum potassium. The rarest of the dyskalemic paralyzes, the incidence of the hyperkalemic variety has been estimated to be 1:500,000.^ ^Known precipitating factors are potassium intake, fasting, hypothermia, infection, stress, rest after exercise, and anesthesia.^ ^The key to successful management is avoidance of triggering factors, vigilant monitoring of potassium, and aggressive treatment of hyperkalemia. We present a case of a 41-year-old male with HyperPP who underwent general anesthesia successfully.

## Introduction

Hyperkalemic periodic paralysis (HyperPP) is a rare form of potassium-associated familial paralysis characterized by episodic flaccid weakness with an increase in serum potassium [1]. The underlying genetic defect is a mutation of the α subunit of the skeletal muscle sodium channel that results in hypopolarization of the muscle membrane [2,3]. During an episode of paralysis, potassium efflux from muscle cells, causing serum potassium to rise on average by 20%. Given the known precipitating factors, appropriate precautions should be taken during the anesthetic management of patients in the perioperative period for future successful outcomes.

## Case presentation

A 41-year-old male with a past medical history of HyperPP, asthma, and hypertension, underwent laparoscopic sigmoid resection after failing conservative management. Neurology was consulted for preoperative evaluation, and the patient was prescribed a high carbohydrate diet as tolerated with a carbohydrate load the day prior to the surgery. The patient was also prescribed acetazolamide 500 mg oral two times daily up to the day of the procedure.

The day prior to the procedure, the patient tolerated bowel prep well and fasted eight hours. Preoperative electrocardiogram (ECG) and laboratory values were normal, including serum potassium of 4.3 mEq/l and serum glucose of 82 mg/dl. The operating room temperature was increased to 72°F, to avoid hypothermia. The patient was transported to the operating room, where standard American Society of Anesthesiologists (ASA) monitors were applied. An intravenous infusion of 5% dextrose in normal saline was initiated in order to maintain adequate glucose levels throughout the operation, which was eventually changed to normal saline to avoid hyperglycemia. All fluids were infused through a warming device, and a forced-air warming blanket was placed on the patient. The patient was pre-medicated with midazolam 2 mg IV, pre-oxygenated and induced with propofol and fentanyl. The patient was intubated and paralyzed using rocuronium, titrated to effect using a twitch monitor. An arterial line was placed in the left radial artery and was used for blood pressure monitoring and frequent arterial blood gas sampling. Serial potassium levels were monitored hourly and ranged from 3.1 to 3.9 mEq/l. Rescue medications such as insulin, glucose, 8.4% sodium bicarbonate, calcium chloride, furosemide, and albuterol were immediately available to treat a potential hyperkalemic crisis.

The duration of the procedure was four hours. The patient was extubated successfully with no complications and transferred to the post-anesthesia care unit. The patient did not exhibit signs of muscle weakness and was discharged home on a post-operative day five in stable condition.

## Discussion

Hyperkalemic periodic paralysis (HyperPP) is a rare form of potassium-associated familial paralysis characterized by episodic flaccid weakness with an increase in serum potassium [1]. In approximately half of the affected individuals, episodes of flaccid muscle weakness begin in the first decade of life, with 25% reporting their first episode at age ten years or older [2,4]. Diagnosis is based on clinical findings and/or the identification of a heterozygous genetic variant in sodium voltage-gated channel alpha subunit 4 (SNC4A).

Three distinct subgroups of this disorder have been described based on potassium concentrations during the episodes of weakness: (1) hypokalemic, (2) normokalemic, and (3) hyperkalemic [3]. The hyperkalemic subgroup has three clinical variants: HyperPP with myotonia, HyperPP without myotonia, and HyperPP with paramyotonia (cold-induced myotonia) [1].

The underlying genetic defect is a mutation of the α subunit of the skeletal muscle sodium channel that results in hypopolarization of the muscle membrane [3,5]. During an episode of paralysis, potassium efflux from muscle cells, causing serum potassium to rise on average by 20%. This relative hyperkalemia depolarizes the muscle membrane sufficiently to prevent activation of sodium channels and propagation of the action potential causing weakness or paralysis [3,4]. Weakness is more pronounced in the proximal muscles of the extremities and trunk, but may progress to affect facial and bulbar muscles; while muscles of respiration are generally spared [3]. Known precipitating factors are excess potassium intake, carbohydrate depletion, prolonged fasting, hypothermia, emotional or physical stress, infection, rest after exercise, and anesthesia [2,3,6].

The anesthetic management of these patients is challenging given the rarity of the condition and the paucity of literature available. Preparation for elective surgery should begin well in advance and include neurology consultation. Baseline electrolytes and ECG should be obtained. Pre-operative potassium reduction using a kaliuretic, such as loop-acting diuretics, thiazides, or carbonic anhydrase inhibitors is recommended [1]. Maintaining normoglycemia can be achieved by minimizing excess fasting (i.e., schedule surgery early in the morning), promoting carbohydrate loading preoperatively and administering dextrose-containing intravenous fluids as needed [3]. Specific intraoperative medications such as depolarizing muscle relaxants (i.e., succinylcholine), cholinesterase inhibitors (e.g., neostigmine), and potassium-containing intravenous fluids could precipitate a paralytic episode and should be avoided [7,8].

Hypothermia should be avoided by ensuring ambient operating room temperature between 72°F and 75°F, using a forced-air warming blanket and a fluid warming device [8,9]. Close monitoring of the patient’s acid-base status and serum potassium levels using an arterial line is recommended [9]. Avoiding acidosis is crucial as this could promote potassium efflux extracellularly which could precipitate paralysis [1]. Examining the ECG for evidence of hyperkalemia such as tall, peaked T waves could assist in early recognition.

Rescue medications should be readily accessible in the event of muscle weakness. Several medications are recommended including: (1) insulin (with glucose if level <200 mg/dL), (2) β2 adrenergic agonists (intravenous or inhaled), (3) loop diuretics, (4) sodium bicarbonate, (5) calcium chloride or gluconate, and (6) epinephrine. Postoperatively, effective pain management and maintenance of electrolyte balance are vital for favorable outcomes and patient throughput.

## Conclusions

Hyperkalemic periodic paralysis (HyperPP) is a rare genetic disorder characterized by episodic flaccid weakness and increased serum potassium. Given the known precipitating factors, appropriate precautions should be taken during the anesthetic management of patients in the perioperative period for a successful outcome. We have presented the anesthetic considerations and management of a 41-year-old male with HyperPP who underwent general anesthesia successfully.

## References

[1] Ashwood EM, Russell WJ, Burrow DD (1992). Hyperkalaemic periodic paralysis and anaesthesia. Anaesthesia.

[2] Weber F, Jurkat-Rott K, Lehmann-Horn F (2003). Hyperkalemic periodic paralysis. GeneReviews®.

[3] Weller JF, Elliott RA, Pronovost PJ (2002). Spinal anesthesia for a patient with familial hyperkalemic periodic paralysis. Anesthesiology.

[4] Miller J, Katz RL (1981). Muscle diseases. Anesthesia and Uncommon Disease: Pathophysiologic and Clinical Correlations, 2nd ed.

[5] Greene SA (2010). Anesthesia for patients with neurologic disease. Top Companion Anim Med.

[6] Charles G, Zheng C, Lehmann-Horn F, Jurkat-Rott K, Levitt J (2013). Characterization of hyperkalemic periodic paralysis: a survey of genetically diagnosed individuals. J Neurol.

[7] Barker MC (2010). Combined spinal/general anesthesia with postoperative femoral nerve block for total knee replacement in a patient with familial hyperkalemic periodic paralysis: a case report. AANA J.

[8] Patangi SO, Garner M, Powell H (2012). Management of a patient with hyperkalemic periodic paralysis requiring coronary artery bypass grafts. Ann Card Anaesth.

[9] Yong SL, Sin TH, Tang EB, Chai MC (2018). Hyperkalaemic periodic paralysis in pregnancy. BMJ Case Rep.

